# Doses *Lactobacillus reuteri* depend on adhesive ability to modulate the intestinal immune response and metabolism in mice challenged with lipopolysaccharide

**DOI:** 10.1038/srep28332

**Published:** 2016-06-21

**Authors:** Kan Gao, Li Liu, Xiaoxiao Dou, Chong Wang, Jianxin Liu, Wenming Zhang, Haifeng Wang

**Affiliations:** 1 Institute of Animal Nutrition, College of Animal Science and Technology, Zhejiang A & F University, Lin’an 311300, Zhejiang Province, P.R. China; 2 College of Animal Science, MOE Key Laboratory of Molecular Animal Nutrition, Zhejiang University, Hangzhou 310029, P.R. China

## Abstract

The objective of this study was to evaluate the modulatory effects of *Lactobacillus reuteri* ZJ617 and ZJ615, which have high and low adhesive abilities, respectively, and *Lactobacillus rhamnosus* GG (LGG) on immune responses and metabolism in mice stimulated with lipopolysaccharide (LPS). Six C57BL/6 mice per group were orally inoculated with ZJ617, ZJ615 or LGG for one week (1 × 10^8^ CFU/mouse) and i.p. injected with LPS (10 mg/kg) for 24 h. Compared with the LPS stimulation group, ZJ615, ZJ617 and LGG significantly decreased TNF-α levels in the sera of mice stimulated by LPS. ZJ615 and LGG significantly down-regulated mRNA levels of cytokines and Toll-like receptors, and suppressed activation of MAPK and NF-κB signaling, while ZJ617 up-regulated anti-inflammatory cytokine IL-10 mRNA levels in the ilea of mice stimulated by LPS. Correlation analysis confirmed that adhesive ability is relative with the immunomodulation in the ilea of mice. There were 24, 7 and 10 metabolites and 10, 9 and 8 major metabolic pathways with significant differences (VIP > 1, *P* < 0.05) between the LPS and ZJ617 + LPS groups, the LPS and ZJ615 + LPS groups, and the ZJ617 + LPS and ZJ615 + LPS groups, respectively. The results indicated that both ZJ617 and ZJ615 could modulate the intestinal immune responses and metabolism in LPS-stimulated mice.

Lactobacilli are probiotics in the gastrointestinal tract that help maintain gut homeostasis^1^. Several reports have shown that the probiotic *Lactobacillus coryniformis* could reduce pro-inflammatory status in mice^2^. *Lactobacillus rhamnosus* OLL2838 had modulatory effects on a mouse model of intestinal immunopathology^3^. Probiotic *Lactobacillus reuteri* ameliorated elevated inflammatory responses in the colon of infected mice and inhibited dysbiosis^4^.

Intestinal *Lactobacillus reuteri* are beneficial microbes that play an important probiotic role in maintaining gut health^5^. A previous study demonstrated that *Lactobacillus reuteri* promotes anti-inflammatory activities in mice with typhlocolitis^6^. *Lactobacillus reuteri* ATCC PTA 6475 decreased TNF-α mRNA level to attenuate intestinal inflammation in mice^7^.

Adhesive ability is important for bacterial function in the intestines of the host. Two high adhesive strains of *Lactobacillus* had anti-inflammatory effects on *Salmonella*-infected intestinal epithelial cells (IEC)^8^. *Lactobacillus rhamnosus* GG (LGG) is a probiotic that has well-documented adhesive properties and beneficial effects on IECs stimulated with endotoxin, via modulating cytokine mRNA expressions^9^,^10^. In our previous study, two *Lactobacillus reuteri* strains were isolated from piglets and identified as ZJ617, with high adhesive ability, and ZJ615 with low adhesive ability, with adhesion indexes of 12.35 ± 0.09 and 1.21 ± 0.14 CFU/cell, respectively, determined by the adherence assays of lactobacillus to an *in vitro* cultured Caco-2 cell monolayer. That is to say, ZJ617 adhesion to Caco-2 cells was an order of magnitude higher than ZJ615^11^. Therefore, the immunomodulatory effects of strains with different adhesive abilities were examined in this study.

Metabolomics can be used in gastrointestinal (GI) tract research to better understand the changes in metabolites in intestinal diseases, such as inflammatory bowel disease, and predict and identify novel biomarkers for the treatment of intestinal diseases^12^. Among the technologies used in metabolomics, gas chromatography/time-of-flight mass spectrometry (GC-TOF-MS) has been widely applied because of its high sensitivity^13^. Previous studies mainly focused on the effects of diet on metabolic activity in animals^13^,^14^, and the effect of colonic microbiota on gut health and immunity^15^,^16^. The overall metabolic phenotypes (metabotypes) reflect myriad functions encoded in host genomes and gut microbiomes. The particular intestinal microbial configurations can promote or prevent inflammatory immune responses that drive metabolic dysfunction^17^. The gut microbiota is pivotal for homeostasis in the intestine, and chronic activation of the innate and adaptive immune system is linked to immunosenescence. Correlations have previously been found between specific components of the microbiota and pro-inflammatory cytokine levels^18^. However, metabolomic studies on host intestines modulation by lactobacilli are limited.

The aims of this study were to investigate the probiotic effects of ZJ617 and ZJ615, which have different adhesive abilities, in an inflammatory mouse model *in vivo*, with LGG as a reference strain, to elucidate the mechanisms of lactobacilli as probiotics in the mammalian intestine. We also aimed to elucidate the metabolic mechanisms via investigating the metabolic profiles in the intestinal contents of *Lactobacilli*-treated mice.

## Results

### TNF-α levels and biochemical measurements in the sera of LAB pre-treated mice after LPS challenge

**Figure 1:**
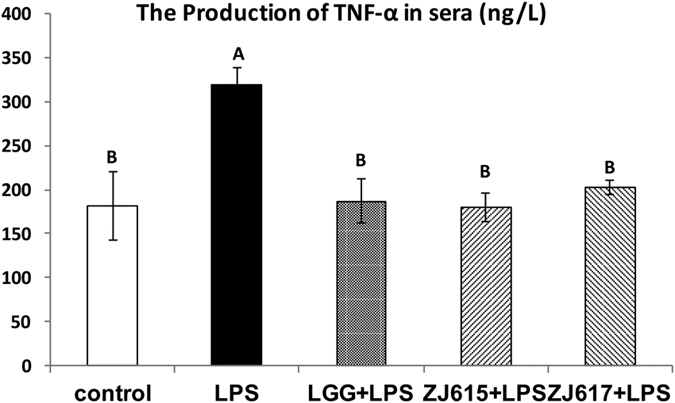
The production of TNF-α in mouse sera. Mice were orally inoculated with lactobacilli for one week and then i.p. injected with LPS for 24 h. Sera were collected from all mice immediately after euthanasia and TNF-α levels in the sera were detected by ELISA. The values are expressed as the mean ± SD (n = 6). The means for TNF-α without a common letter differ significantly (*P* < 0.05).

**Figure 2:**
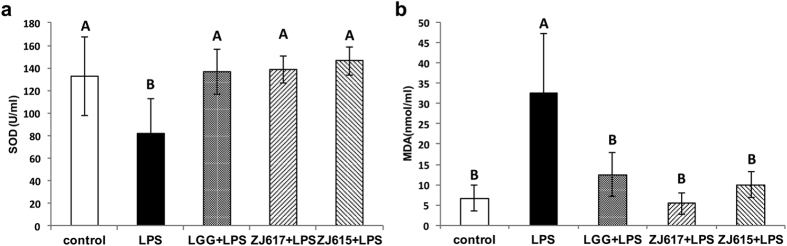
Biochemical measurements of SOD and MDA in mouse sera. Mice were orally inoculated with lactobacilli for one week and then i.p. injected with LPS for 24 h. Sera were collected from all mice immediately after euthanasia. SOD (**a**) and MDA (**b**) in sera were detected by ELISA. The values are expressed as the mean ± SD (n = 6). The means for each biochemical index without a common letter differ significantly (*P* < 0.05).

To evaluate the probiotic effect of ZJ617 and ZJ615 on mice after LPS stimulation, the level of TNF-α and biochemical measurements (SOD and MDA) in serum were assessed. Compared with the LPS groups, LGG, ZJ617 and ZJ615 could significantly decreased TNF-α levels in the sera of mice stimulated by LPS (Fig. 1). Additionally, LGG, ZJ617 and ZJ615 restored SOD levels (Fig. 2a) and significantly decreased MDA levels in sera of mice stimulated by LPS (*P* < 0.05, Fig. 2b).

### The relative expression of cytokines and TLRs in the ilea of LAB pre-treated mice after LPS challenge

**Figure 3:**
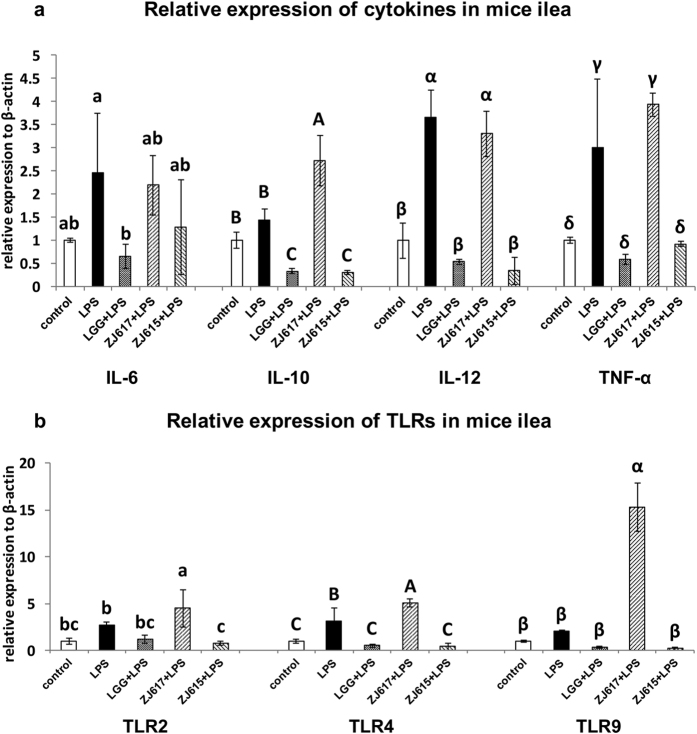
The relative expression of cytokines (**a**) and TLRs (**b**) in the ilea of mice pre-treated with LAB and challenged with LPS. Mice were orally inoculated with lactobacilli for one week and then stimulated with LPS via i.p injection. Ileal tissues from six mice in each group were collected after euthanasia and examined by qRT-PCR. The values are expressed as the mean ± SD (n = 6). The means for each cytokine or TLR without a common letter differ significantly (*P* < 0.05).

To investigate the probiotic effect of ZJ617 and ZJ615 on the ileum of mice stimulated by LPS, the relative mRNA level of cytokines (IL-6, IL-10, IL-12 and TNF-α) and TLRs (TLR2, TLR4 and TLR9) in ilea were measured. Compared with the LPS groups, LGG and ZJ615 significantly decreased, while ZJ617 significantly increased, IL-10, IL-12 and TNF-α mRNA in the ilea of mice stimulated by LPS (Fig. 3a). TLR2, TLR4 and TLR9 mRNA levels were significantly decreased by LGG and ZJ615 and were significantly increased by ZJ617 in the ilea of mice stimulated by LPS compared with LPS group (Fig. 3b, *P* < 0.05).

### The expression of cell signaling molecules in the ilea of LAB pre-treated mice stimulated by LPS

**Figure 4:**
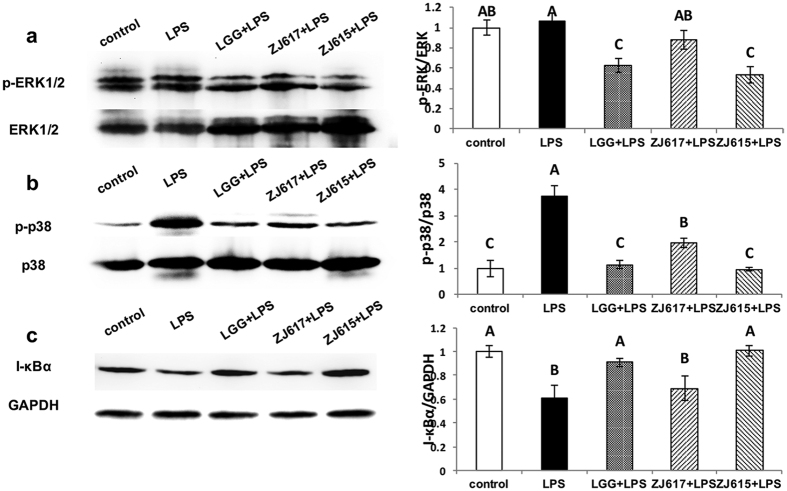
The expression of cell signaling molecules in the ilea of mice pre-treated with LAB and challenged with LPS. Mice were orally inoculated with lactobacilli for one week and then stimulated with LPS via i.p injection. Ileal tissues from six mice in each group were collected after euthanasia and examined by western blot. Phosphorylation of ERK1/2 and p38 MAPK and expression of I-κBα were detected and represented as a ratio of p-ERK/ERK (**a**), p-p38 MAPK/p38 MAPK (**b**) and I-κBα/GAPDH (**c**). The gray scale values are expressed as the mean ± SD (n = 6). The means for each signaling factor without a common letter differ significantly (*P* < 0.05).

To explore the mechanism of immunomodulation of ZJ617 and ZJ615 on the ilea of mice stimulated by LPS, the activation of cell signaling pathways (p38MAPK, ERK1/2 and NF-κB) in the ilea was assessed. LGG and ZJ615 significantly decreased phosphorylation of ERK1/2 in the ilea of mice stimulated by LPS, while ZJ617 had no significant effects on ERK1/2 signaling compared with the LPS groups (Fig. 4a). LGG, ZJ617 and ZJ615 significantly decreased phosphorylation levels of p38 MAPK in the ilea of mice stimulated by LPS (Fig. 4b). LGG and ZJ615 restored I-κBα to normal levels, while ZJ617 had no significant effects on I-κBα (*P* > 0.05, Fig. 4c).

### The expression of cell signaling molecules in the ilea of LAB pre-treated mice stimulated by LPS verified by IHC

**Figure 5:**
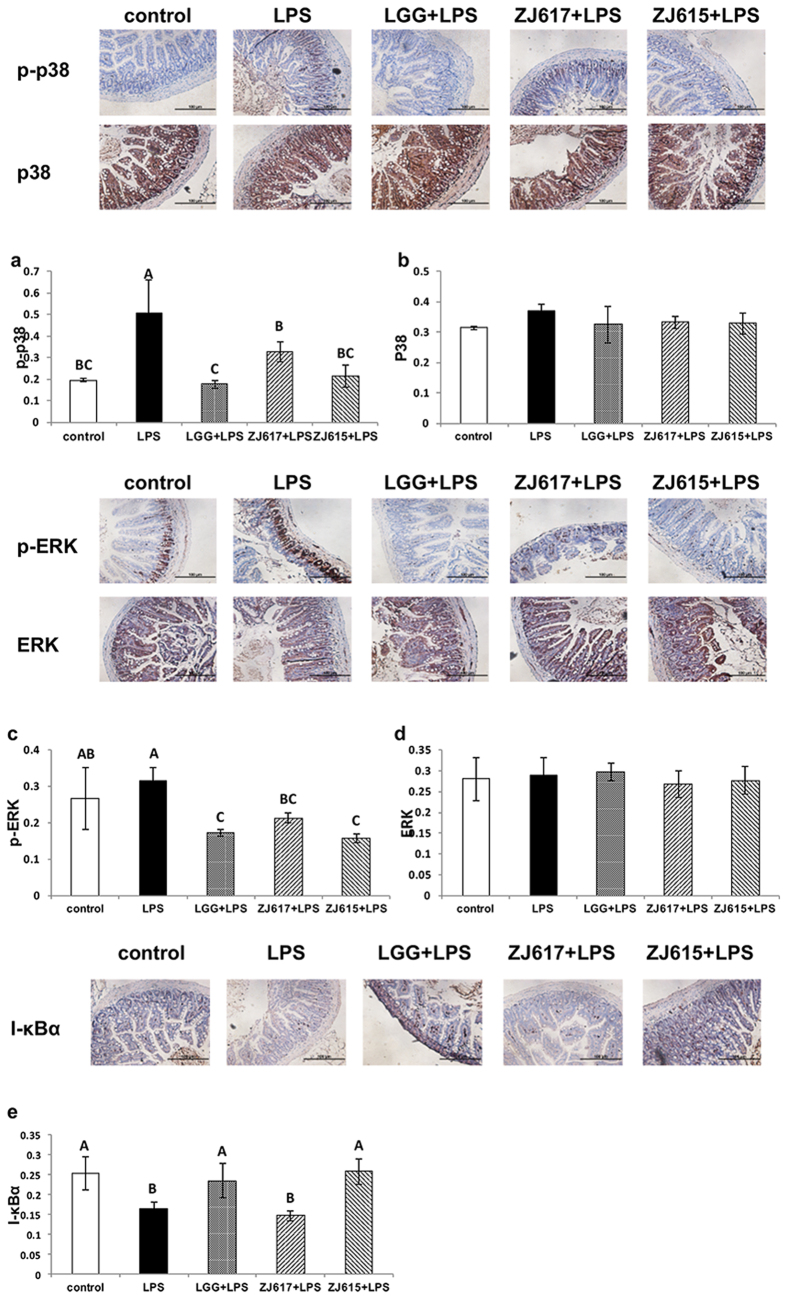
The expression of cell signaling molecules in the ilea of mice pre-treated with LAB and challenged with LPS. Mice were orally inoculated with lactobacilli for one week and then stimulated with LPS via i.p injection. Ileal tissues from six mice in each group were collected after euthanasia and examined by IHC. Phosphorylation of ERK1/2 and p38 MAPK and expression of I-κBα were detected and represented as p-p38 (**a**), p38 (**b**), p-ERK (**c**), ERK (**d**) and I-κBα (**e**). The ratio of positive-stained cells/all cells is expressed as the mean ± SD (n = 6). The means for each signaling factor without a common letter differ significantly (*P* < 0.05).

To confirm the immunomodulatory effects of ZJ617 and ZJ615, the level of cell signaling molecules (phospho-p38, p38, phospho-ERK1/2, ERK1/2 and I-κBα) in the ilea was verified by IHC. All IHC samples were analyzed using Image Pro-Plus (Cambridge, UK). Consistent with the results of western-blot analysis, LGG and ZJ615 significantly decreased the phosphorylation levels of ERK1/2 in the ilea of mice stimulated by LPS, while ZJ617 had no significant effects on ERK1/2 signaling compared with the LPS only group (Fig. 5a). Additionally, LGG, ZJ617 and ZJ615 significantly decreased phosphorylation of p38 MAPK (Fig. 5b). While LGG and ZJ615 increased I-κBα levels to normal in the ilea (*P* < 0.05), ZJ617 had no significant effects on NF-κB signaling (*P* > 0.05, Fig. 5c).

### Correlation of bacterial adhesion and immunomodulation among lactobacillus strains

**Figure 6:**
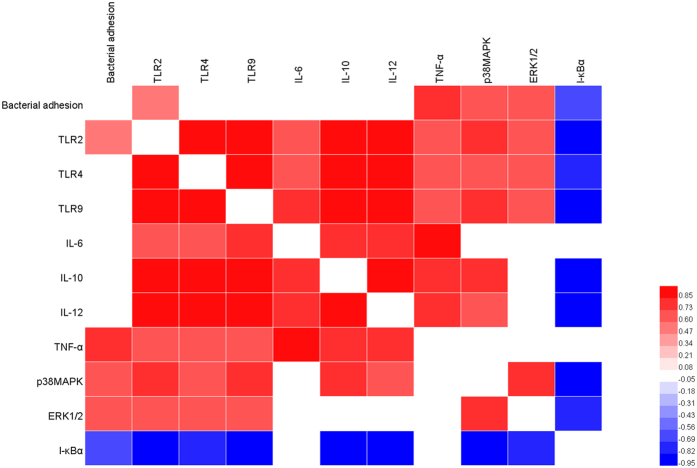
Spearman’s correlation analysis of bacterial adhesion and immune responses related factors. The red represents a significant positive correlation (*P* < 0.05), the blue represents a significant negative correlation (*P* < 0.05), and the white shows that the correlation was not significant (*P* > 0.05).

The adhesive ability of three lactobacillus strains to the ileum mucosa was determined with mice (Fig. S1B). The number of ZJ617 adhering to the ileal mucosa was significantly higher than others. To study the correlation between the adhesive ability and immunomodulation of these lactobacillus strains, we performed Spearman’s correlation analysis (Fig. 6). Bacterial adhesion was positively correlated with pro-inflammatory cytokine TNF-α, and inflammatory related signalling p38MAPK and ERK1/2, while negatively correlated with NF-κB negative regulator I-κBα. In addition, bacterial adhesion was positively correlate with TLR2. There findings indicated that adhesive ability was responsible for the ileal immune responses toward LPS stimulation.

### Metabolite profiling of the intestinal contents of mice

**Figure 7:**
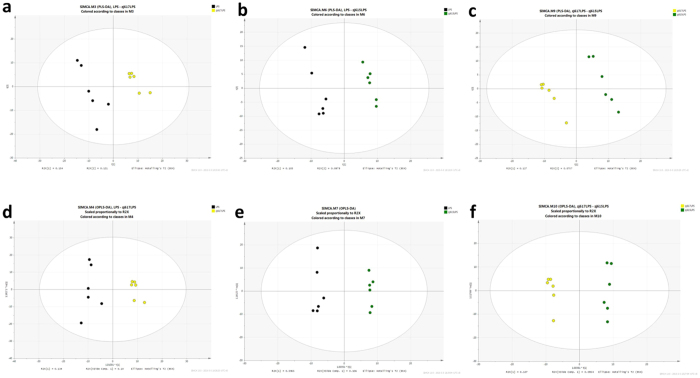
PLS-DA (**a–c**) and OPLS-DA (**d–f**) score map derived from the GC/MS metabolite profiles of intestinal contents. Black represents contents from the LPS group, yellow represents contents from the ZJ617 + LPS group, and green represents contents from the ZJ615 + LPS group (n = 6).

Small molecular weight metabolites were extracted from the intestinal contents of ZJ617- or ZJ615-treated mice stimulated by LPS, or the corresponding mice stimulated with LPS alone. Based on the LECO/Fiehn Metabolomics Library, a total of 883 metabolite peaks were determined (Fig. S3). The PCA analysis of the GC-TOF-MS metabolic profiles of the three groups showed clusters with no significant differences among the LPS, ZJ617 + LPS and ZJ615 + LPS groups in a 3D-PCA score plot (Fig. S4). To obtain a higher level of group separation and a better understanding of the variables responsible for classification, partial least squares discriminant analysis (PLS-DA) and orthogonal projections to latent structures discriminant analysis (OPLS-DA) were applied (Fig. 7). Clear separation and discrimination were found among the LPS, ZJ617 + LPS and ZJ615 + LPS groups, suggesting that the OPLS-DA model can be used to identify the differences among three groups (Fig. 7d–f). All significantly different metabolites (VIP > 1, *P* < 0.05) were identified, followed by a statistical comparison of the peak values among the LPS-stimulated group, the ZJ617 + LPS group and the ZJ615 + LPS group. In the intestinal contents of the LPS-stimulated mice, the levels of 24 metabolites were significantly changed compared to those of the mice in the ZJ617 + LPS group (Table 1). In the intestinal contents of the LPS-stimulated mice the levels of 8 metabolites were significantly changed compared to those of mice in the ZJ615 + LPS group (Table 2). In the intestinal contents of the mice in the ZJ617 + LPS group, the levels of 10 metabolites were significantly changed compared to those of mice in the ZJ615 + LPS group (Table 3).

### Changes in the levels of metabolites and metabolic pathways in mice

The fold change (FC) value was used to assess the quantity among the LPS, ZJ617 + LPS and ZJ615 + LPS groups. Compared with the LPS group, the ZJ617 + LPS group had 20 metabolites with higher concentrations out of 24 significantly different metabolites in the intestinal contents (Table 1). The higher concentration metabolites in the ZJ617 + LPS group included 4 carbohydrate metabolites, glucose (FC = 5 × 10^−9^), D-gluconic acid (FC = 0.0869), D-arabitol (FC = 1.3 × 10^−8^) and isomaltose (FC = 1.592 × 10^−6^), 4 lipid metabolites, mannitol (FC = 2.12 × 10^−7^), 2-methylglutaric acid (FC = 0.410), 5-dihydrocortisone (FC = 0.103), maleic acid (FC = 0.478), and others. Compared with the LPS group, the ZJ615 + LPS group had 6 metabolites with higher concentrations out of 7 significantly different metabolites in the intestinal contents (Table 2). The higher concentration metabolites in the ZJ615 + LPS group included 2 amino acid metabolites, valine (FC = 0.290) and phenylalanine (FC = 0.276), 2 lipid metabolites, arachidonic acid (FC = 0.241) and 2-methylglutaric acid (FC = 0.294), and other organic compounds. Compared with the ZJ615 + LPS group, the ZJ617 + LPS group had 8 metabolites with higher concentrations out of 10 significantly different metabolites in the intestinal contents (Table 3), which included 4 carbohydrate metabolites, such as glucose (FC = 6.020), xylitol (FC = 7.771), D-gluconic acid (FC = 5.611) and isomaltose (FC = 7.459), and other organic compounds. The KEGG pathway analysis of significantly different metabolites among the three groups identified 10 metabolic pathways, such as biosynthesis of antibiotics and galactose metabolism, which were the major differentially expressed metabolic pathways between the LPS and ZJ617 + LPS groups (Table 4). Similarly, 9 metabolic pathways, including protein digestion and absorption and biosynthesis of amino acids, were the key different metabolic pathways between the LPS and ZJ615 + LPS groups (Table 5). Eight main metabolic pathways, including carbohydrate digestion and absorption and galactose metabolism, were identified as different between the ZJ617 + LPS and ZJ615 + LPS groups (Table 6).

## Discussion

We established an inflammatory C57BL/6 mouse model *in vivo* with LGG, a known probiotic, as a reference strain to investigate the probiotic effects of ZJ617, with high adhesive ability, and ZJ615, with low adhesive ability, which were previously isolated from piglets^11^.

*Lactobacillus plantarum* CECT 7315/7316 could attenuate inflammation in LPS-induced mice^19^. *Lactobacillus reuteri* CRL1101 down-regulated the level of cytokines (IL-1β, IL-6 and TNF-α) in the sera of mice stimulated by LPS^20^. Consistent with these reports, in this study, both ZJ617 and ZJ615 significantly down-regulated TNF-α in the sera of mice stimulated by LPS, indicating that ZJ617 and ZJ615 had protective effects on mice challenged with an endotoxin.

We found that pretreatment with ZJ617, ZJ615 or LGG significantly increased anti-oxidant SOD activity and decreased MDA levels in the serum of LPS-challenged mice (Fig. 2). Consistent with these results, pretreatment with *Lactobacillus casei* Zhang also up-regulated SOD activity and down-regulated MDA levels in serum of mice stimulated by LPS^21^. *Lactobacillus plantarum* NDC 75017 showed protective effects against LPS stimulation, which restored the level of SOD and MDA to normal^22^. Our findings indicate that ZJ617 and ZJ615 had anti-inflammatory and anti-oxidative effects on mice challenged by LPS.

A previous report^23^ showed that oral administration of *Lactobacillus casei* CRL431 could decrease mRNA levels of TLR2, TLR4 and TLR9 in the intestine of mice challenged with *Salmonella* and also significantly decreased the mRNA levels of inflammatory cytokines (TNF-α and IFN-γ) and anti-inflammatory cytokine IL-10. Consistent with the above report, oral administration of ZJ615 or LGG significantly decreased mRNA levels of TLRs (TLR2, TLR4 and TLR9), inflammatory cytokines (IL-6, IL-12 and TNF-α) and anti-inflammatory cytokine IL-10 in the ilea of mice after LPS challenge compared with the no lactobacillus LPS group (Fig. 3). These findings suggest that ZJ615 and LGG may exert a probiotic effect on the intestine of mice via down-regulating mRNA levels of TLRs (TLR2, TLR4 and TLR9) and cytokines to attenuate intestinal inflammation.

The effects of high-adhesive ZJ617 on the ilea were different from the effects of ZJ615 and LGG. Oral administration of ZJ617 increased mRNA levels of TLRs and triggered higher anti-inflammatory cytokine IL-10 mRNA expression in the ileum of mice compared with the LPS only group. The results suggest that ZJ617 could interact with intestine directly and more efficiently. Consistent with our findings, several studies reported that *Lactobacillus reuteri* protected the intestine from external stimuli by maintaining a certain amount in the intestinal tract, thus triggering intestinal cytokines and chemokines to maintain homeostasis^24^. *Lactobacillus reuteri* significantly increased anti-inflammatory IL-10 production in mice with typhlocolitis^6^.

NF-κB signaling and MAPK signaling are important pathways involved in the modulation of host inflammation triggered by stimuli^25^,^26^. *Lactobacillus brevis G-101* could attenuate intestinal inflammation by inhibiting NF-κB and MAPK activation^27^. Consistent with the above reports, ZJ615 and LGG could suppressed phosphorylation of p38 MAPK, ERK1/2 and activation of NF-κB signaling induced by LPS, indicating that ZJ615 and LGG could exert a probiotic effect on the ilea via regulating the NF-κB and MAPK pathways. Interestingly, ZJ617 also suppressed MAPK activation but had no effect on NF-κB activation. These results deserve further study.

In the present study, correlation analysis revealed that the adhesive ability of three lactobacillus strains was responsible for the ileal immune responses toward LPS stimulation in mice. Previous study reported that TLR2 as an inflammatory mediator in GI tract could activate MAPK and NF-κB signaling to respond to environmental stimuli^23^. Here, we hypothesized that, compared with high adhesive ability strain ZJ617, low adhesive ability strain ZJ615 and LGG might suppressed TLR2 expression on the ileal mucosa to attenuate inflammation induced by LPS injection, which was verified in the present study and was consistent with the results in our previous *in vitro* study^9^.

Metabolomic analyses, in which thousands of small molecular weight metabolites can be analyzed in different types of samples, offer a promising approach to evaluate functional status in the interaction between microbiota and the host intestines^16^. GC-TOF-MS is a widely used approach in metabolomics for its high resolution and sensitivity^13^,^28^. Metabolomic studies evaluating the effects of *Lactobacilli* on the intestines of the host are limited. To our knowledge, this is the first study to use a metabolomic approach to investigate the functional mechanisms of the interaction between *Lactobacilli* and the metabolism of the host. The aim was to identify metabolites that were significantly altered in the intestinal contents, thus providing a better understanding of the metabolic pathways involved in this interaction.

In this metabolomic study of intestinal contents, the OPLS-DA model could be used to identify all significantly different metabolites (VIP > 1, *P* < 0.05) among the three groups (Fig. 7d–f). By peak value comparison among the LPS, ZJ617 + LPS and ZJ615 + LPS groups, we found 24, 7 and 10 metabolites were significantly changed between the LPS and ZJ617 + LPS group, the LPS and ZJ615 + LPS group, or the ZJ617 + LPS and ZJ615 + LPS group, respectively. A previous study showed that higher counts of intestinal *Lactobacillus* species are associated with higher glucose content, and specific fermentative groups and species of lactobacilli appear at different glucose levels^29^. In this study, glucose (FC = 5 × 10^−9^), D-gluconic acid (FC = 0.0869) and N-acetyl-D-galactosamine (FC = 0.113) were significantly higher in the ZJ617 + LPS group than the LPS group (Table 1). Glucose (FC = 6.020) and gluconic acid (FC = 5.611) were significantly higher in the ZJ617 + LPS group than the ZJ615 + LPS group (Table 2). Glucose and its derivatives showed no significant differences between the ZJ615 + LPS group and the LPS group. Galactose (FC = 0.394) was significantly higher in the ZJ615 + LPS group cpmpared to the ZJ617 + LPS group (Table 3). These findings suggest that ZJ617 increases glucose contents in the intestinal lumen, while ZJ615 enhances galactose contents in the intestinal. Fermentation of lactose by lactobacilli in the intestinal lumen was accompanied by the release of free galactose^30^.

Interestingly, our findings showed that the essential amino acids L-phenylalanine (FC = 0.276) and L-valine (FC = 0.290) were altered in the ZJ615 + LPS group compared to the LPS group (Table 2), which indicates that ZJ615 could regulate amino acid utilization in the intestines of the host. Consistent with our findings, previous research demonstrated that *Lactobacillus* GG is associated with a reduction in endotoxemia and dysbiosis, indicating that changes in metabolite are related to amino acid metabolism in the host^10^. The metabolic pathway analysis based on the KEGG database showed that metabolic pathways such as biosynthesis of antibiotics and mineral absorption were involved in the LPS, ZJ617 + LPS and ZJ615 + LPS groups (Table 4, 5, 6, [Table t5], [Table t6]). For biosynthesis of antibiotics, *Lactobacillus reuteri* DSM 17938 was reported to have potential antimicrobial effects against the major gastric and enteric bacterial pathogens and rotavirus by modulating metabolic pathways^31^. A previous study showed that *Lactobacillus helveticus* could affect intestinal mineral absorption^32^, which is consistent with our findings. Combined with the metabolic pathway analysis, we found that glucose and its derivatives may be potential biomarkers, which require further research.

In conclusion, two *Lactobacillus reuteri* strains with different adhesive abilities exerted immunoregulatory effects on the intestines of LPS-challenged mice by regulating TLR2, TLR4 and TLR9 and modulating the activation of NF-κB and MAPK signaling, thus regulating cytokine levels in the intestine to alleviate inflammation. Adhesive ability of lactobacillus strains is one of factors responsible for the ileal immune responses toward environmental stimuli. The metabolomic analysis of intestinal contents using GC-TOF-MS indicated that several metabolites were significantly changed among the LPS, ZJ617 + LPS and ZJ615 + LPS groups, including glucose and its derivatives, galactose, amino acids such as phenylalanine and valine, and also metabolic pathways, such as the biosynthesis of antibiotics, ABC transporters and mineral absorption, among others. The results from the present study provide insight into the mechanism of interaction between lactobacilli and the host, indicating that both high-adhesive ZJ617 and low-adhesive ZJ615 had modulatory effects on the intestinal immune response and metabolism in LPS-stimulated mice.

## Methods

### Mice and reagents

C57BL/6 mice (20 ± 2 g, 6–8 weeks old) were purchased from the Model Animal Research Center of Nanjing University (Nanjing, China). Animal care was performed according to protocols approved by the Animal Care and Use Committee in Zhejiang A & F University. LPS and streptomycin were purchased from Sigma-Aldrich (St.Louis, MO, USA). Antibodies specific to phospho-p38 MAPK, p38 MAPK, phospho-ERK1/2, ERK1/2, I-κBα and glyceraldehyde-3-phosphate dehydrogenase (GAPDH) were obtained from Cell Signaling Technology (Danvers, MA, USA). HRP (horseradish peroxidase)-conjugated secondary antibodies were obtained from Jackson ImmunoResearch (West Grove, PA, USA).

### Experimental treatment and sample collection

ZJ617 (high adhesive ability) and ZJ615 (low adhesive ability) were previously isolated from piglets^11^. LGG was a gift from Prof. Jinru Chen at the University of Georgia. In this study, bacteria were anaerobically grown at 37 °C in de Man, Rogosa, and Sharp broth (MRS broth) (Hope Bio, Qingdao, Shandong, China) for 18 h and then stored at −80 °C. Mice were kept at a constant temperature of 26 ± 2 °C with 12 h light–dark cycles and provided with free access to water and a standard diet. The mice were randomly assigned to five groups (n = 6): two of them, designed as the control and LPS groups, received PBS. The other three treatments were named LGG + LPS, ZJ617 + LPS and ZJ615 + LPS, and mice in these group were orally inoculated with, respectively LGG, ZJ617 or ZJ615 suspended in sterile PBS at a concentration of 1 × 10^8^ CFU/ml daily for one week. The number of lactobacilli in the discharged feces (n = 6) and adhering to the ileal mucosa after 24 h LPS injection (n = 8) was evaluated (Fig. S1). Inflammation was induced in mice with an intraperitoneal (i.p.) injection of 10 mg/kg LPS, with an exception for the control group mice which received an i.p. injection of sterile PBS instead. LPS did not cause mortality in the next 24 h determined in a pilot study (Fig. S2). Blood samples were collected by cardiac puncture 24 h after the LPS challenge. The blood was allowed to clot at 4 °C for 2 h and centrifuged at 3000 g for 10 min at 4°C to obtain serum samples that were stored at −80 °C. Intestinal contents and ileal tissues were collected at 24 h after the LPS challenge and stored at −80 °C until use.

### Serum cytokine and biochemical analysis

Inflammatory cytokine TNF-α and biochemical measurements (superoxide dismutase, SOD; malondialdehyde, MDA) in serum samples were evaluated by a mouse TNF-α ELISA kit (Nanjing Jiancheng Bioengineering Institution, Nanjing, China) according to the manufacturer’s instruction.

### RNA extraction and real-time quantitative PCR

Total RNA from ileal tissue was extracted using TRIzol (Invitrogen, CARLSBAD, CA, USA) in liquid nitrogen according to the manufacturer’s instruction. Reverse transcription was carried out using a reverse transcription kit (TaKaRa Bio, Shiga, Japan), and cDNA was stored at −20 °C until use. Quantitative real-time PCR (qRT-PCR) was performed using SYBR® Premix Ex Taq (TaKaRa Bio), according to the manufacturer’s instructions. The reaction was conducted using the Mx3000P^TM^ system (Agilent, Palo Alto, CA, USA) that was programmed for denaturation at 95°C for 30 s, followed by 40 cycles of 95°C for 5 s and 60°C for 30 s. The sequences of the PCR primers^33^ were as follows: β-actin (5′-TGGAATCCTGTGGCATCCATGAAAC-3′, 5′-TAAAACGCAGCTCAGTAACAGTCCG-3′); IL-6 (5′-ACCACGGCCTTCCCTACTT-3′, 5′-CACAACTCTTTTCTCATTTCCAC-3′); IL-10 (5′-CCCTTTGC TATGGTGTCCTT-3′, 5′-TGGTTTCTCTTCCCAAGACC-3′); IL-12 (5′-GGAAGCACGGCAGCAGAAT-3′, 5′-GGCGGGTCTGGTTTGATG-3′); TNF-α (5′-TGGGAGTAGACAAGGTACAACCC-3′, 5′-CATCTT CTCAAAATTCGAGTGACAA-3′); TLR2 (5′-AAGATGTCGTTCAAGGAGGTGCG-3′, 5′-ATCCTCT GAGATTTGACGCTTTG-3′); TLR4 (5′-GGTGTGAAATTGAGACAATTGAAAAC-3′, 5′-GTTTC CTGTCAGTACCAAGGTTGA-3′); TLR9 (5′-ATCTCCCAACATGGTTCTCCG-3′, 5′-GATACGGTTGGA GATCAAGGAG-3′).

Data were analyzed using the Mx3000P^TM^ system software. The relative quantification of gene expression with β-actin as an internal standard was determined by the cycle threshold (Ct) method as follows: ∆∆Ct = [Ct(target gene) – Ct(housekeeping gene)] treatment – [Ct(target gene) – Ct(housekeeping gene)] control. The final data were derived from the formula 2^−∆∆Ct^.

### Western blot

Ileal tissues were lysed using lysis buffer (Sigma-Aldrich) in liquid nitrogen according to the manufacturer’s instructions. The concentration of protein in samples was determined by Bradford’s method^34^. Total protein samples separated with SDS-PAGE, transferred to PVDF membranes, blocked and incubated with primary antibody at 4°C overnight. After incubation with the HRP-conjugated secondary antibody, the blot was developed with ECL (Millipore, Merck KGaA, Darmstadt, Germany). The optical density of the bands was measured using ImageJ software (National Institutes of Health, Bethesda, MD, USA).

### Immunohistochemistry

Ileal tissues were fixed in formalin, embedded in paraffin, and cut into 4 μm sections according to previous methods^35^. Deparaffinized and rehydrated sections were blocked with 10% normal goat serum for 1 h. Sections were incubated with primary antibodies overnight at 4 °C and then with HRP-conjugated secondary antibody for 1 h. The diaminobenzidine-HRP detection system was used, and sections were then counterstained with hematoxylin, dehydrated and cover-slipped. Positive IHC staining^36^ was determined with a microscope (ECLIPSE Ti, Nikon Corp., Tokyo, Japan).

### Preparation of intestinal contents for GC-TOF-MS

Twenty mg ileal contents from each mouse was extracted with 0.4 mL methanol-chloroform (V_methanol_ : V_chloroform_ = 3 : 1) with the addition of 30 μL L-2-chlorophenylalanine (1 mg/ml stock in ddH_2_O) as an internal standard. The sample was homogenized in a ball mill for 3 min at 65 Hz and then centrifuged at 12,000 rpm for 15 min at 4 °C. Supernatants were transferred to a GC-TOF-MS glass vial, and 20 μl of each sample was added to one glass vial as a mixed sample for quality control. After metabolite extracts were dried in a vacuum concentrator without heating for 1.5 h, 60 μl microliter methoxymethyl amine salt (dissolved in pyridine, 20 mg/mL) was added into the dried metabolite extracts for 20 min at 80 °C. After the addition of 80 μL BSTFA (containing 1% TCMS, v/v), each extract sample was incubated at 70 °C for 1 h and then subjected to analysis via GC-TOF-MS.

### GC-TOF-MS analysis

Based on a previous study^28^, GC-TOF-MS analysis was performed with an Agilent 7890 gas chromatograph system linked to a Pegasus HT time-of-flight mass spectrometer (LECO, St. Joseph, MI, USA). The system employed a DB-5 column with 5% diphenyl and 95% dimethyl polysiloxane (J&W Scientific, Folsom, CA, USA). The GC column temperature was programmed to rise from 50 to 330 °C at a rate of 10 °C/min. One microliter of sample was injected in splitless mode. The energy was −70 eV in electron impact mode. Data were acquired in full-scan mode with an m/z range of 30–600 at a 20 spectra/sec velocity.

### Metabolomics data analysis

Chroma TOF 4.3X software from the LECO Corporation and the LECO-Fiehn Rtx5 database were used for raw peaks extraction, baseline filtering and calibration of the baseline, peak alignment, deconvolution analysis, peak identification and integration of the peak area based on the previous study^37^. The SIMCA-P 13.0 software package (Umetrics, Umea, Sweden) was used for principal component analysis (PCA), partial least squares discriminant analysis (PLS-DA) and orthogonal projections to latent structures-discriminant analysis (OPLS-DA). After unit variance (UV) scaling in the data process, PCA was applied to show the origin data set. The PLS-DA model validated by 200 permutation tests was used to obtain a higher level of data separation. Furthermore, OPLS-DA was performed to obtain maximal covariance among the data. To refine the analysis, the first principal component of variable importance projection (VIP) was obtained. The VIP values exceeding 1.0 were first selected as changed metabolites, then assessed by Student’s T test (T-test), *P* > 0.05, and variables were discarded between two comparison groups. The fold change (FC) of metabolites was obtained by comparing mean peak values between two groups. Obtained metabolites were validated by searching in the Kyoto Encyclopedia of Genes and Genomes (KEGG), and each metabolite was cross-linked with pathways in the KEGG.

### Statistics

The data from qRT-PCR, western-blots and IHC are expressed as the mean ± standard deviation (SD) of the replicates. The statistical significance was evaluated using one-way analysis of variance (ANOVA, general linear model), followed by Duncan’s multiple range test using the SAS program (SAS Institute, INC, USA). Differences are considered significant if *P* < 0.05. Spearman’s correlation analysis was conducted in the Graphpad Prism 5.0 software (GRAPHPAD Software, San Diego, CA, USA).

## Additional Information

**How to cite this article**: Gao, K. *et al*. Doses *Lactobacillus reuteri* depend on adhesive ability to modulate the intestinal immune response and metabolism in mice challenged with lipopolysaccharide. *Sci. Rep.* **6**, 28332; doi: 10.1038/srep28332 (2016).

## Supplementary Material

Supplementary Information

## Figures and Tables

**Table 1:** List of significantly changed metabolites in the LPS group and the ZJ617 + LPS group.

**Metabolite Name**	**Similarity**	**R.T.**^a^	**Mass**	**VIP**^b^	***P*** **value**^c^	**FC**^d^
Glucose	947	17.6	160	2.07537	0.01882	0.000000005
Phosphate	857	10.2295	299	1.6685	0.04329	0.622410586
Dihydroxyacetone	789	10.0093	73	1.74531	0.049958	10.905710000
Linoleic acid methyl ester	701	19.5305	136	1.85993	0.039649	0.000000007
Mannitol	686	18.0156	217	2.05745	0.020158	0.000000212
Cellobiotol	640	25.0222	204	1.79564	0.047739	0.024980229
N-alpha-Acetyl-L-ornithine	626	18.5482	174	1.68145	0.041292	5.255360417
L-Allothreonine	566	10.5319	146	2.13237	0.014876	0.011969541
Adenosine	538	24.0068	236	1.80949	0.03999	4.385818670
3-Hydroxypalmitic acid	483	20.5048	97	2.58686	2.97E-05	0.199293761
Malonic acid	344	12.2685	327	1.78901	0.02715	0.566624153
DL-Anabasine	330	11.9323	242	1.96328	0.012173	0.158024143
N-Acetyl-D-galactosamine	327	19.2005	221	2.11035	0.005269	0.112974527
2-Methylglutaric Acid	318	12.1692	86	1.83937	0.021907	0.409576937
Gluconic acid	293	18.6894	293	2.47033	0.000201	0.086936749
Atrazine-2-hydroxy	291	17.2002	140	1.6619	0.044334	0.307408421
5-Dihydrocortisone	279	28.5782	221	1.77299	0.044077	0.103250509
3-Methylamino-1,2-propanediol	273	11.6638	84	1.68952	0.04008	0.244076542
Acetol	269	13.2167	57	1.89974	0.016642	7.203885285
Maleic acid	246	10.6076	341	1.86114	0.019886	0.478113079
2-hydroxypyridine	232	6.83189	136	1.83894	0.021948	0.468394215
D-Arabitol	194	15.7746	36	1.97175	0.027488	0.000000013
5,6-dihydrouracil	147	13.204	265	2.13435	0.004513	0.115115987
Isomaltose	134	25.6871	533	2.30141	0.006788	0.000001592

^a^R.T. represented retention time.

^b^VIP = variable importance projection, metabolite (VIP > 1) was listed in table.

^c^*P* values were calculated according to Student’s T-test.

^d^FC represented as the fold change of the peak intensity for the LPS group against the ZJ617 + LPS group (n = 6).

**Table 2:** List of significantly changed metabolites in the LPS group and the ZJ615 + LPS group.

**Metabolite Name**	**Similarity**	**R.T.**^a^	**Mass**	**VIP**^b^	***P*** **value**^c^	**FC**^d^
Valine	929	9.44723	144	1.57663	0.037152	0.290305
Arachidonic acid	854	21.9473	91	1.98539	0.043569	0.24063
Phenylalanine	774	13.9178	120	1.24562	0.048931	0.276223
2,3-Dihydroxypyridine	750	10.8217	240	1.55255	0.048411	0.377064
Digitoxose	391	14.5243	73	1.72961	0.022967	7.440897
2-Methylglutaric Acid	318	12.1692	86	1.6088	0.020425	0.293636
Thymidine	118	10.2904	341	1.56786	0.023045	0.393048

^a^R.T. represented retention time.

^b^VIP = variable importance projection, metabolite (VIP > 1) was listed in table.

^c^*P* values were calculated according to Student’s T-test.

^d^FC represented as the fold change of the peak intensity for the ZJ617 + LPS group against the ZJ615 + LPS group (n = 6).

**Table 3:** List of significantly changed metabolites in the ZJ617 + LPS group and the ZJ615 + LPS group.

**Metabolite Name**	**Similarity**	**R.T.**^a^	**Mass**	**VIP**^b^	***P*** **value**^c^	**FC**^d^
Glucose	947	17.6	160	2.24825	0.029406	6.01968
Xylitol	653	15.6597	217	2.05975	0.019828	7.77052
Galactose	577	17.7864	110	1.132	0.003196	0.39415
Sarcosine	529	8.38449	116	1.78932	0.024567	36.60145
DL-Anabasine	330	11.9323	242	2.07843	0.013412	6.10723
Gluconic acid	293	18.6894	293	2.23567	0.000876	5.61108
2-ketobutyric acid	233	7.81917	157	1.58586	0.022761	3.83510
Cortisone	165	25.5915	69	1.72329	0.049621	0.55608
5,6-dihydrouracil	147	13.204	265	2.56683	0.005456	442163.48470
Isomaltose	134	25.6871	533	2.0911	0.00799	7.45914

^a^R.T. represented retention time.

^b^VIP = variable importance projection, metabolite (VIP > 1) was listed in table.

^c^*P* values were calculated according to Student’s T-test.

^d^FC represented as the fold change of the peak intensity for the ZJ617 + LPS group against the ZJ615 + LPS group (n = 6).

**Table 4:** Metabolic pathways identified from significantly changed metabolites in the LPS group and the ZJ617 + LPS group.

	**Metabolic pathways**									
	**Biosynthesis of antibiotics**	**ABC transporters**	**Mineral absorption**	**Carbon metabolism**	**beta-Alanine metabolism**	**Galactose metabolism**	**Pentose phosphate pathway**	**Starch and sucrose metabolism**	**Parkinson’s disease**	**Pyrimidine metabolism**
LPS-ZJ617 + LPS	D-Glucose (FC^a^ = 5 × 10^−9^)	Phosphate (FC = 0.622)	Phosphate (FC = 0.622)	Dihydroxyacetone (FC = 10.906)	Maleic acid (FC = 0.478)	D-Glucose (FC = 5 × 10^−9^)	D-Glucose (FC = 5 × 10^−9^)	D-Glucose (FC = 5 × 10^−9^)	Phosphate (FC = 0.622)	Maleic acid (FC = 0.478)
	D-Gluconic acid (FC = 0.087)	D-Glucose (FC = 5 × 10^−9^)	D-Glucose (FC = 5 × 10^−9^)	D-Gluconic acid (FC = 0.087)	5,6-Dihydrouracil (FC = 0.115)	N-Acetyl-D-galactosamine (FC = 0.113)	D-Gluconic acid (FC = 0.087)	Isomaltose (FC = 1.59 × 10^−6^)	Adenosine (FC = 4.386)	5,6-Dihydrouracil (FC = 0.115)
	N-Acetylornithine (FC = 5.255)	Mannitol (FC = 2.12 × 10^−7^)								

^a^FC represented as the fold change of the peak intensity for the LPS group against the ZJ617 + LPS group (n = 6).

**Table 5 t5:** Metabolic pathways identified from significantly changed metabolites in the LPS group and the ZJ615 + LPS group.

	**Metabolic pathways**								
	**Biosynthesis of antibiotics**	**ABC transporters**	**Mineral absorption**	**Central carbon metabolism in cancer**	**Biosynthesis of amino acids**	**2-Oxocarboxylic acid metabolism**	**Cyanoamino acid metabolism**	**Aminoacyl-tRNA biosynthesis**	**Protein digestion and absorption**
LPS-ZJ615+LPS	L-Phenylalanine (FC^a^ = 0.276)	L-Phenylalanine (FC = 0.276)	L-Phenylalanine (FC = 0.276)	L-Phenylalanine (FC = 0.276)	L-Phenylalanine (FC = 0.276)	L-Phenylalanine (FC = 0.276)	L-Phenylalanine (FC = 0.276)	L-Phenylalanine (FC = 0.276)	L-Phenylalanine (FC = 0.276)
	L-Valine (FC = 0.290)	L-Valine (FC = 0.290)	L-Valine (FC = 0.290)	L-Valine (FC = 0.290)	L-Valine (FC = 0.290)	L-Valine (FC = 0.290)	L-Valine (FC = 0.290)	L-Valine (FC = 0.290)	L-Valine (FC = 0.290)

^a^FC represented as the fold change of the peak intensity for the LPS group against the ZJ615 + LPS group (n = 6).

**Table 6 t6:** Metabolic pathways identified from significantly changed metabolites in the ZJ617 + LPS group and the ZJ615 + LPS group.

	**Metabolic pathways**							
	**Biosynthesis of antibiotics**	**ABC transporters**	**Mineral absorption**	**Carbohydrate digestion and absorption**	**Glycine, serine and threonine metabolism**	**Galactose metabolism**	**Pentose phosphate pathway**	**Starch and sucrose metabolism**
ZJ617+LPS-ZJ615 + LPS	D-Glucose (FC^a^ = 6.020)	D-Glucose (FC = 6.020)	D-Glucose (FC = 6.020)	D-Glucose (FC = 6.020)	2-Ketobutyric acid (FC = 3.835)	D-Glucose (FC = 6.020)	D-Glucose (FC = 6.020)	D-Glucose (FC = 6.020)
	2-Ketobutyric acid (FC = 3.835)	Xylitol (FC = 7.771)	D-Galactose (FC = 0.394)	D-Galactose (FC = 0.394)	Sarcosine (FC = 36.601)	D-Galactose (FC = 0.394)	D-Gluconic acid (FC = 5.611)	Isomaltose (FC = 7.459)
	D-Gluconic acid (FC = 5.611)							

^a^FC represented as the fold change of the peak intensity for the ZJ617 + LPS group against the ZJ615 + LPS group (n = 6).
