# Supplementary material for: Doses Lactobacillus reuteri depend on adhesive ability to modulate the intestinal immune response and metabolism in mice challenged with lipopolysaccharide
Source: Sci Rep. 2016 Jun 21;6:28332. doi: 10.1038/srep28332 (PMC4915000; doi:10.1038/srep28332)
Supplement: Supplementary Information [file srep28332-s1.doc]

**Supplementary Information**

**Doses *Lactobacillus* *reuteri* depend on adhesive ability to modulate the intestinal immune response and metabolism in mice challenged with lipopolysaccharide**

Kan Gao^1^, Li Liu^1^, Xiaoxiao Dou^1^, Chong Wang^1^, Jianxin Liu^2^, Wenming Zhang^2^, Haifeng Wang1[[1]](#footnote-2)*

^1^*Institute of Animal Nutrition, College of Animal Science and Technology, Zhejiang A & F University, Lin’an 311300, Zhejiang Province, P.R. China;*

*^2^College of Animal Science, MOE Key Laboratory of Molecular Animal Nutrition, Zhejiang University, Hangzhou 310029, P.R. China.*

*Corresponding author

Dr. Hai-Feng Wang

Phone: +86-571-63743313

Fax: +86-571-63741751

E-mail: hfwang@zafu.edu.cn

**Supplementary Figure S1. The number of ZJ615, ZJ617 and LGG cells in feces (A) and adhering to the ileal mucosa (B) from mice.** Mice were orally inoculated with a 10^8^ CFU dose of LGG, ZJ617 or ZJ615 for one week, fresh feces from mice at the 4^th^ day after the first day of LAB inoculation were collected (A), and the number of lactobacilli adhering to the ileal mucosa collected by slaughter at 24 h after LPS injection on day 8 (B). Data are expressed as the mean ± SD (n=6 for fecal samples and n=8 for intestinal tract). The means for each strain without a common letter differ significantly (*P* < 0.05).


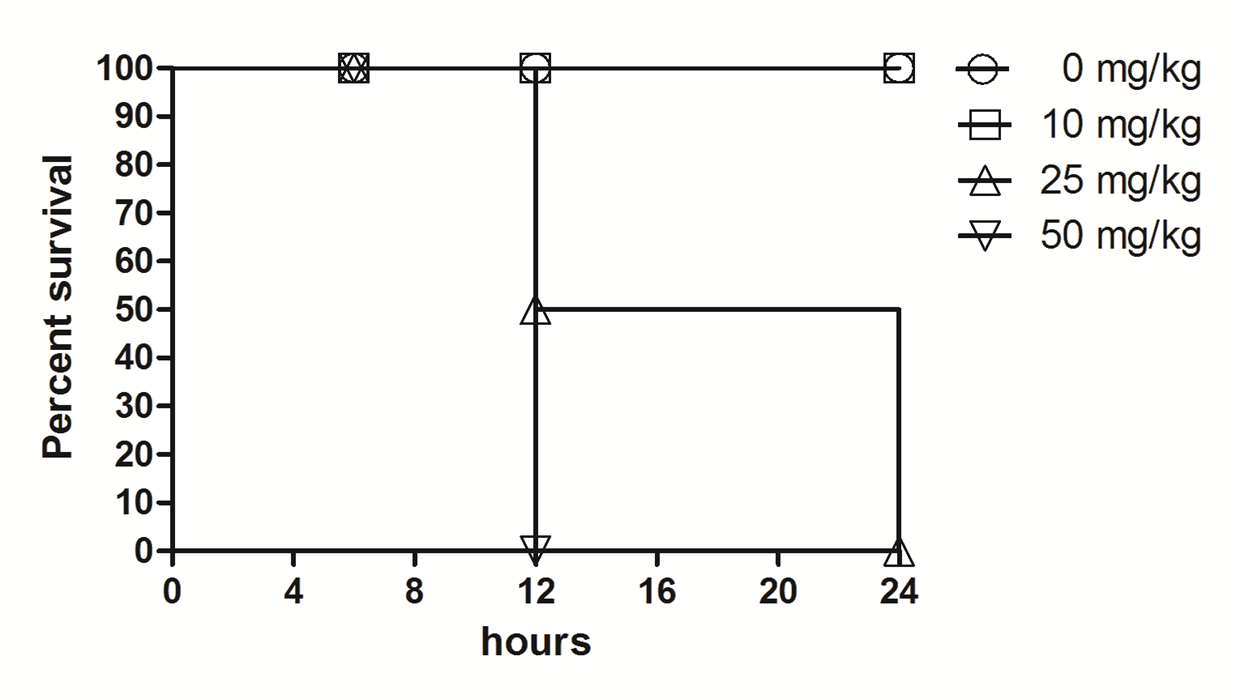


**Supplementary Figure S2. Detection of lethality in LPS-stimulated mice.** Mice were divided into 4 groups (n=6) with 0 μg/mouse, 200 μg/mouse, 500 μg/mouse, or 1000 μg/mouse stimulation respectively.


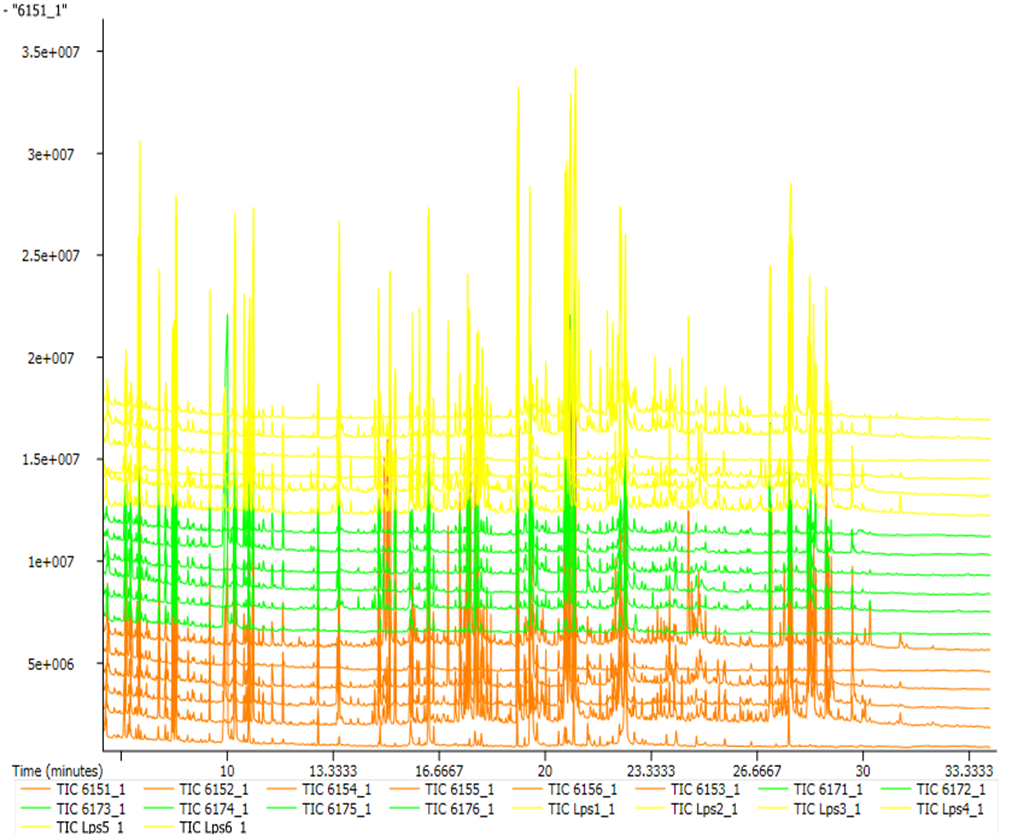


**Supplementary Figure S3. GC-TOF-MS TIC chromatograms of intestinal contents of the LPS group (yellow), the ZJ617+LPS group (green) and the ZJ615+LPS group (orange).**


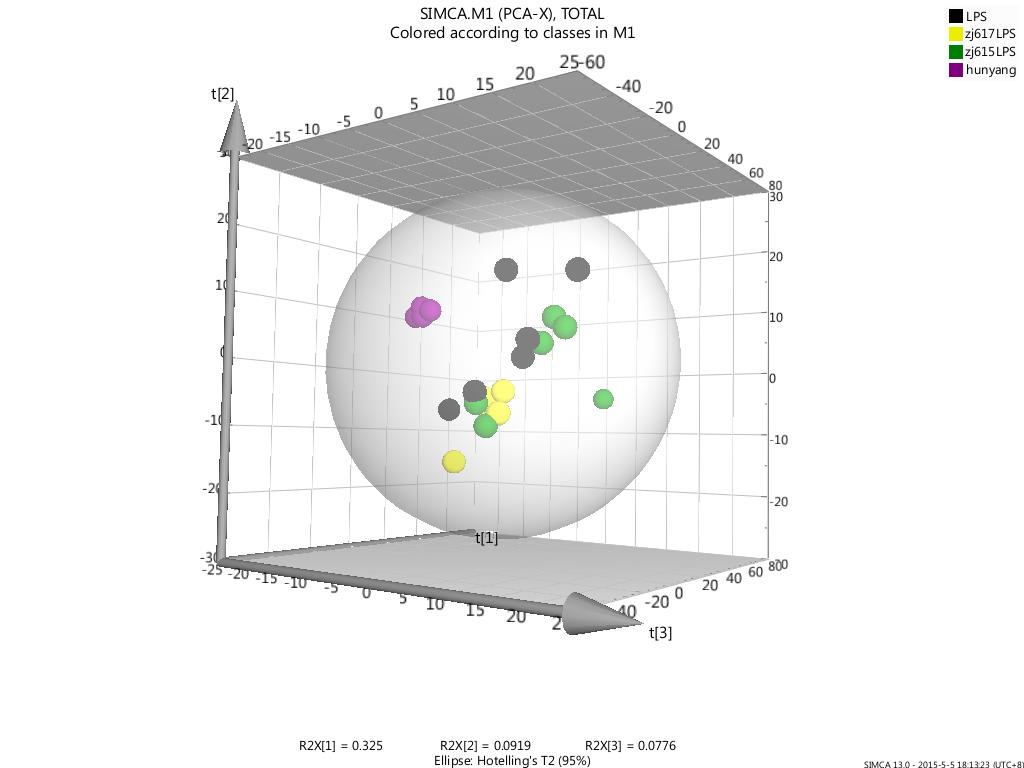


**Supplementary Figure S4. PCA 3D score map derived from the GC-MS metabolite profiles of intestinal contents**. Black dots represents contents from the LPS group, yellow dots represents contents from the ZJ617+LPS group, green dots represents contents from the ZJ615+LPS group, and purple dots represents mixed contents. (n=6)

1. ^^ Institute of Animal Nutrition, College of Animal Science, Zhejiang A & F University, Lin’an, 311300, Zhejiang Province, P.R. China. [↑](#footnote-ref-2)
